# Mindfulness vs. sleep education during autologous hematopoietic cell transplantation for multiple myeloma: Feasibility of a randomized controlled pilot study

**DOI:** 10.1016/j.conctc.2025.101540

**Published:** 2025-08-18

**Authors:** Elisabeth C. Henley, Hannah A. Liphart, Keayra J. Morris, Iwalola Awoyinka, Michael R. Irwin, Erin S. Costanzo, Diana Winston, Anita D'Souza, Melinda Stolley, Binod Dhakal, Meera Mohan, Marcelo C. Pasquini, Steven W. Cole, Erin S. Doerwald, Peyton C. Bendis, Kelly E. Rentscher, Meredith E. Rumble, Aniko Szabo, Sridhar Rao, Jennifer M. Knight

**Affiliations:** a Department of Psychiatry and Behavioral Medicine, Medical College of Wisconsin, Milwaukee, WI, USA; b Department of Medicine, Medical College of Wisconsin, Milwaukee, WI, USA; c Department of Psychiatry and Biobehavioral Sciences, University of California Los Angeles, Los Angeles, CA, USA; d Cousins Center for Psychoneuroimmunology, Semel Institue for Neuroscience and Human Behavior at University of California Los Angeles, Los Angeles, CA, USA; e Department of Psychiatry, University of Wisconsin School of Medicine and Public Health, Madison, WI, USA; f Department of Medicine, Division of Hematology-Oncology, Medical College of Wisconsin, Milwaukee, WI, USA; g Hello Wellness Therapy, Santa Fe, NM, USA; h Institute of Health and Equity, Division of Biostatistics, Medical College of Wisconsin, Milwaukee, WI, USA; i Versiti Blood Research Institute, Department of Cell Biology, Neurobiology, and Anatomy, Department of Pediatrics, Division of Pediatric Hematology/Oncology/Transplantation, Medical College of Wisconsin, Milwaukee, WI, USA; j Department of Microbiology & Immunology, Medical College of Wisconsin, Milwaukee, WI, USA

**Keywords:** Multiple myeloma, Hematopoietic stem cell transplantation, Mindfulness-based intervention, Peri-transplant, Feasibility, Sleep

## Abstract

**Background:**

Sleep disturbance is common in patients receiving hematopoietic stem cell transplantation (HCT). Mindfulness-based interventions (MBIs) can improve sleep quality during and following cancer treatment by reducing treatment-related symptoms and enhancing immune function.

**Methods:**

We conducted a randomized controlled pilot study investigating the feasibility of implementing Mindfulness Awareness Practices for Insomnia (MAP-I) in patients with multiple myeloma (MM) undergoing autologous HCT. Patients were randomized to receive either MAP-I or a Sleep Health Education (SHE) intervention, both consisting of six videos viewed pre-HCT and three virtual sessions in the two weeks post-HCT. Feasibility was assessed by meeting an enrollment rate of 35% and a retention rate of 85%.

**Results:**

We screened 120 patients; 54 (45%) were deemed ineligible and 42 (35%) declined participation. Twenty-four of the 66 eligible patients approached were enrolled into the study (36.4% enrollment rate) and were randomized to either MAP-I or SHE. Seven patients completed the study (29.2% retention rate). Most participants who withdrew consent cited feeling overwhelmed or too sick to continue post-HCT. Amendments were iteratively implemented to increase enrollment and retention rates including addition of a study incentive, modifications to the video timeline, and earlier introduction of the mindfulness instructor.

**Conclusion:**

Study results detail challenges and opportunities in retaining patients with MM in a virtual MBI sleep intervention during the peri-transplant period. While enrollment met feasibility criteria, most patients felt too overwhelmed or sick in the peri-transplant period to complete the intervention and associated study tasks. Future research should investigate MBIs at other time points throughout HCT.

**Trial registration:**

NCT04271930, 2/17/2020.

## Highlights

• RCT of a mindfulness-based intervention in stem cell transplant recipients.
• Feeling overwhelmed and/or sick in the peri-transplant period impacted retention.
• Iterative changes made to the protocol design that targeted retention are outlined.
• Discussion addresses barriers to retention during the peri-transplant period.

## 1 Introduction

Hematologic malignancies are often treated with hematopoietic cell transplantation (HCT), and while survival rates have increased, patients often suffer from significant physical and psychological demands [1,2]. Multiple myeloma (MM) is one of the most common hematologic malignancies and accounts for approximately 67% of all autologous HCTs per year, establishing it as one of the most frequent indications for this treatment [3,4]. While HCT promotes the possibility of remission, MM remains incurable, and patients undergo a lengthy hospital stay along with potentially distressing post-HCT sequelae [[5], [6], [7]].

HCT recipients frequently report symptoms of anxiety, depression, and sleep disturbance in the peri-transplant period, a window encompassing the pre-transplant conditioning phase through hospitalization [8,9]. Eighty-two percent of patients experience significant sleep disruption while hospitalized for HCT, with many reporting ongoing sleep troubles in the year following transplant [[10], [11], [12]]. Sleep impairment often results from physical side effects of treatment, irregular sleep patterns, and psychological distress [10,11]. While nursing care and other disturbances can adversely impact inpatient sleep, 68% of hospitalized HCT recipients report that stress contributes to their sleep difficulties [[12], [13], [14]].

Sleep disruption correlates with impaired physical processes, including slowed recovery, increased infections, and disease progression [8], all of which negatively impact successful post-HCT recovery [15,16]. Sleep disturbance is a commonly overlooked side effect of HCT and is rarely discussed between patients and providers [10]. To combat sleep problems, HCT recipients are often prescribed pharmacologic sleep aids [17,18]. However, each new drug added to a patient's regimen increases the risk of hazardous drug-drug interactions and mortality, a particular concern for post-HCT patients who may manage over a hundred pills per week [[17], [18], [19], [20], [21]]. Therefore, behavioral interventions that target sleep quality are essential to reduce psychological distress and ultimately maximize HCT outcomes.

One of the most well-documented therapies to reduce distress in oncology patients is mindfulness-based interventions (MBIs) [[22], [23], [24], [25]]. Mindfulness has been described as maintaining attention to the present moment while remaining non-judgmental [22,26]. Notably, MBIs have been found to improve sleep quality and immune function while reducing psychological distress during and following cancer treatment [[27], [28], [29]]. Mindfulness Awareness Practices for Insomnia (MAP-I) is an evidenced-based MBI that teaches the integration of mindfulness into everyday life, and specifically emphasizes the practice of mindfulness at bedtime [30,31]. When tested in older adults, MAP-I has demonstrated significant improvement in sleep disturbance, depression, and fatigue [30].

This is the first known study to investigate the feasibility of a virtual MBI (MAP-I) within a randomized control trial (RCT) in the HCT population. Prior work supports the feasibility of delivering an MBI to HCT recipients in the peri-HCT period [5,23], with Bauer-Wu et al. (2008) citing a recruitment rate of 87% and a completion rate of 78.9% [5]. However, both were single-arm studies with the MBI delivered in-person, an intervention strategy with scalability challenges and limitations for patients with travel and access limitations, or issues secondary to being immunocompromised. Here, we examine the feasibility of a virtual MBI (MAP-I) designed to target sleep quality in the peri-transplant period compared with a time and attention Sleep Hygiene Education (SHE) control. We assessed feasibility by meeting both a 35% enrollment and 85% retention rate and hypothesized that MAP-I would be feasible to implement in patients with MM undergoing autologous HCT.

## 2 Methods

### 2.1 Study design

This two-armed, non-blinded pilot RCT investigated the feasibility of a peri-transplant MAP-I intervention in MM patients receiving their first autologous HCT. Secondary outcomes included sleep quality, inflammatory biomarkers, patient-reported sleep-associated behavioral symptoms (depression, anxiety, and fatigue), and clinical outcomes, and will be reported elsewhere. Procedures were reviewed and approved by the Medical College of Wisconsin's (MCW) Institutional Review Board (IRB). Several amendments were made to improve enrollment and retention. Herein, we describe the final study design as implemented following the amendments and additional information regarding timing and details of each amendment. Table 1 presents when data was collected from participants.

**Table 1:** Study assessments and time points.

Study Assessment/Testing	Baseline (T1; Day −42 ± 14)	T2 (Day −2 ± 2	T3 (Day +14 ± 2	T4 (Day +60 ± 7)	T5 (Day +100 ± 14)	Clinical Follow-Up (2 years)
Patient Demographics	X					
Disease Characteristics	X					
Transplant Characteristics	X	X				
Mindfulness Conceptualization	X	X	X	X	X	
Treatment Expectancy	X					
Patient Reported Outcomes (PROs)	X	X	X	X	X	
Biospecimen Collection	X	X	X	X	X	
Actigraphy Collection	X	X	X	X	X	
Clinical Outcomes		X	X	X	X	X
Participant Stipend		X	X		X	

### 2.2 Eligibility criteria

Patients 18 years of age or older with MM undergoing their first autologous HCT were eligible for enrollment. Additional inclusion criteria were as follows: 1 year since initiation of systemic anti-myeloma therapy, no progression or relapse of myeloma prior to HCT, Karnofsky Performance Score ≥70. Exclusion criteria included prior autologous HCT, the presence of coexisting amyloidosis, and the presence of known obstructive sleep apnea.

### 2.3 Recruitment, enrollment, and randomization

**Fig. 1:**
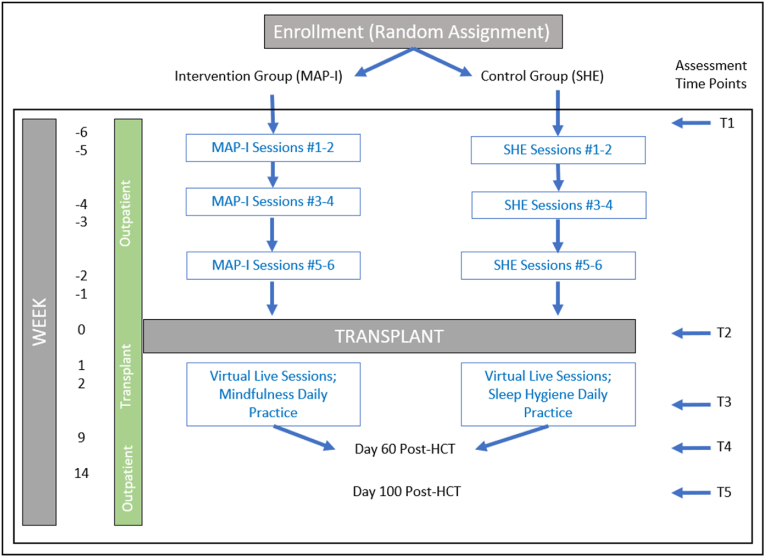
Treatment schema.

Patients were selected from the MCW bone marrow transplant and cellular therapy weekly patient review list. Eligible patients were provided with a study brochure and were briefly introduced to the study by their treating oncologist during an outpatient pre-transplant appointment. Following this brief introduction, a clinical research coordinator (CRC) approached interested patients and sought their consent. Eligible and interested patients signed an MCW IRB-approved informed consent form. After consent was obtained and eligibility confirmed, the CRC randomized participants in a 1:1 allocation ratio via permutated block assignment with random block sizes to receive the MAP-I intervention or a SHE intervention. Patients were consented 2 months (±1 month) prior to HCT, and the study concluded at T5, with a follow-up period of up to 2 years post-transplant (Fig. 1). All participants were provided a stipend 3 times throughout the study (see, Table 1).

### 2.4 Interventions

MAP-I. The MAP-I intervention is curriculum-based and consists of 6 videos delivered via the Teachable website. MAP-I was created by the Mindful Awareness Research Center at the University of California Los Angeles (UCLA) and was adapted for patients undergoing HCT. The MAP-I intervention for HCT recipients maintains the mindfulness exercises that are in the original MAP-I program delivered in older adults but tailors the implementation and practice of the mindfulness exercises for use in hospital settings. Additionally, MAP-I for HCT recipients includes an introductory video that addresses specific sleep challenges commonly experienced during HCT. In addition to the 6 videos, the MAP-I intervention included 3 virtual live sessions with a mindfulness instructor post-transplant. Participants were provided a study iPad to access the intervention videos. MAP-I focuses on treating insomnia symptoms by including the practice of mindfulness before bedtime and during night-time sleep awakenings. It also includes a daily body scan. MAP-I participants were instructed to watch 1–2 sessions per week in the 4–6 weeks prior to HCT (Fig. 1). Participants completed daily bedtime mindfulness practices beginning with 5 min and increasing to 20 min over the 4-6-week intervention period. Participants completed their daily mindfulness practice by following guided mediations via the UCLA Mindful App on the iPad. In the 10–14 days post-HCT, participants were instructed to continue daily mindfulness practice and could re-watch any MAP-I video sessions (Fig. 1).

In addition to the MAP-I course, participants were virtually introduced to a certified mindfulness instructor upon randomization to the MAP-I group. The mindfulness instructor served as the primary contact for MAP-I participants. To facilitate better understanding and daily practice of mindfulness, the mindfulness instructor held live virtual individual or group discussion sessions 2–3 times in the 10–14 days post-HCT (Fig. 1). Attendance, while not mandatory, was encouraged, and the number of sessions attended was recorded.

**SHE.** The SHE intervention consisted of 6 1-h video sessions and 3 virtual live sessions with a CRC. Participants were provided a study iPad to access the SHE videos. The SHE intervention serves as a time and attention control to prevent any confounding effects of nonspecific treatment elements in the evaluation of MAP-I (29). SHE was designed to provide education about sleep problems and factors that may exacerbate sleep disturbance. Participants in the SHE group watched 1–2 sessions per week in the 4–6 weeks pre-HCT (Fig. 1). In addition to the SHE videos, participants met virtually with a CRC 2–3 times in the 10–14 days post-HCT who answered questions and was available for general discussion (Fig. 1). Similar to MAP-I, participants were encouraged to attend, and attendance at each session was recorded.

### 2.5 Qualitative assessment

Participants completed an exit interview at withdrawal or study completion. All participants were asked for feedback on study design, if they found the videos helpful, and what time of day they preferred to watch the videos. Participants who chose to withdraw were asked to indicate why, suggest areas for intervention adjustments, and if they would have stayed on the study if their suggestions were implemented. Participant feedback was used to develop amendments for improved enrollment and retention rates.

### 2.6 Amendments

**Fig. 2:**
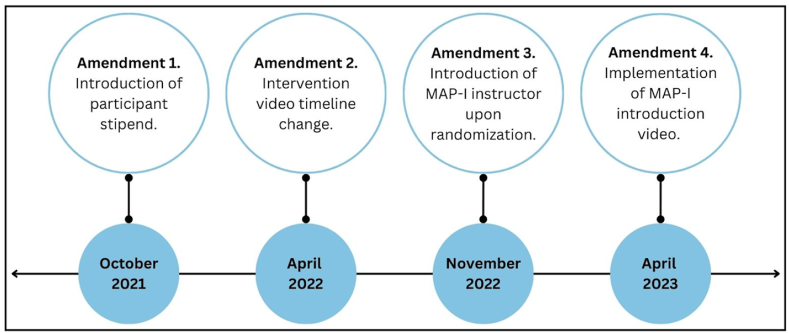
Study amendments timeline.

A total of 4 amendments were made to address enrollment and retention challenges (Table 2). Due to COVID-19, recruitment began virtually in February 2021. In May 2021, when on-site restrictions lifted, patients were recruited in-person. Amendment 1 was implemented in October 2021 which introduced the participant stipend (amendment 1, Fig. 2). In April 2022, amendment 2 altered the MAP-I/SHE video timeline; participants initially viewed 2 videos pre-HCT and 4 videos in the 2–3 weeks post-HCT. With amendment 2, the timeline changed so that all 6 videos were viewed pre-HCT (amendment 2, Fig. 2). This modification provided participants more time to complete the videos and learn mindfulness skills before hospitalization. Amendment 3 was implemented in November 2022 and established introduction of the mindfulness instructor to MAP-I participants upon randomization, providing participants with an ongoing point of contact regarding their practice (amendment 3, Fig. 2). Lastly, in April 2023, a brief video from the mindfulness instructor was included in the introduction e-mail to MAP-I participants to provide an overview of the intervention and detail potential benefits of mindfulness in the peri-transplant period (amendment 4, Fig. 2). For more details about amendments please refer to Table 2.

**Table 2:** Study amendments.

Amendment	Description	Reason
Amendment 1: Addition of Participant Stipend	Participants were awarded 3 $50 Amazon gift cards over the course of the study.	Increase enrollment and retention rates and encourage completion of study activities.
Amendment 2: Expand Eligibility Criteria and Study Timeline Change	Expanded inclusion criteria to encompass both inpatient and outpatient autologous HCT recipients. Changed video timeline from 2 videos pre-HCT and 4 videos during hospitalization to all 6 videos pre-HCT.	Increase enrollment and retention rates. Many participants were withdrawing immediately post-HCT, reporting feeling too overwhelmed to continue. The video timeline change aimed to reduce participant workload post-HCT and maximize mindfulness learning and practice time.
Amendment 3: Addition of MAP-I Person of Contact and Expansion of Video Viewing Schedule	Implemented early contact with the mindfulness teacher via an introduction E-mail to establish connection and a direct avenue for participant questions during MAP-I or SHE video viewing.	Increase retention rate. Many participants had questions as they were learning to meditate. Creating a direct line of communication between participants and the mindfulness instructor helped facilitate questions as they arose. An additional goal was to decrease study participation burden by increasing the time duration participants were allowed to watch MAP-I or SHE videos.
Amendment 4: Mindfulness Instructor Introduction Video	In addition to the introduction E-mail, the mindfulness instructor created a brief video that highlighted the importance of mindfulness and meditation in the peri-transplant period.	Increase retention rate. The goal was to have the mindfulness instructor provide MAP-I participants with an overview of mindfulness and meditation, why mindfulness is beneficial, and why an MBI in the HCT setting may be helpful for reducing sleep disturbance and psychological distress.

### 2.7 Measures

**Demographics.** Demographic information included age at transplant, sex, race, ethnicity, body mass index, smoking status in the 3 months prior to HCT, Karnofsky Performance Score at transplant, medication usage, employment status, and clinical response pre-transplant. Collection occurred at T1 (Table 1).

**Activity Logs.** All participants were given sleep logs to record their daily sleep experience. The sleep log was concurrent with actigraphy collection and occurred T1-T5 over a 7-day period (Table 1). All participants were asked to complete a MAP-I or SHE Weekly Video Log to record the date and time participants watched each video. MAP-I participants were asked to complete a Mindfulness Practice Log to record their daily meditation practices. SHE participants were given a SHE Practice Log to record their daily sleep hygiene practices. All activity logs were used to assess patient adherence to study protocol.

**Biomarkers & Clinical Outcomes.** Inflammatory biomarkers collected included peripheral cytokines, C-reactive protein, and peripheral endocannabinoids. Additional biospecimen samples included whole blood for transcriptome profiling and DNA methylation. Clinical outcomes were assessed through chart reviews and included engraftment syndrome, neutrophil engraftment, platelet engraftment, infections, treatment response, treatment-related mortality, progression-free survival, and overall survival. Methods and results for these biomarkers and clinical outcomes will be reported separately.

### 2.8 Primary outcome

**Feasibility.** Here, we report on the primary outcome of the feasibility of implementing a MAP-I intervention and associated data collection among MM patients undergoing their first autologous HCT. We assess feasibility based on enrollment and retention rates, with a proposed 35% enrollment and 85% retention rate to indicate feasibility based on prior MBI interventions in cancer patients [32], and behavioral interventions in HCT [33].

### 2.9 Secondary outcomes

We assessed select patient-reported outcomes, inflammatory biomarkers, sleep quality, and clinical endpoints as secondary outcomes; data will be reported elsewhere.

### 2.10 Analytic plan

The goal was to recruit a sample size of 20 participants (10 participants per arm); this was based on previous functional genomics studies that successfully evaluated inflammatory biomarkers and psychosocial correlates in similar sample sizes [[34], [35], [36]]. To assess feasibility, the enrollment rate was determined by the number of eligible patients randomized to the trial divided by the number of eligible patients screened. The retention rate was determined by the percentage of participants who continued study participation through day 100 post-HCT, regardless of the number of intervention videos watched, virtual sessions attended, or study activities completed (Table 1 and Fig. 1).

## 3 Results

**Fig. 3:**
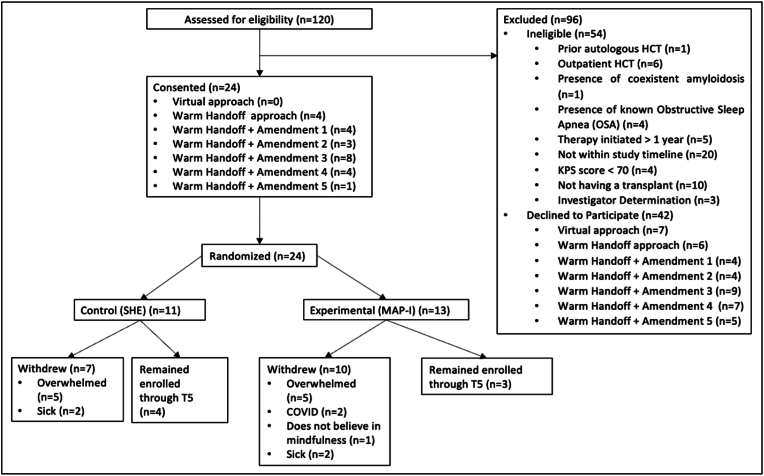
CONSORT flow diagram.

Patients were recruited from February 2021 to August 2023, with 120 MM patients soon to undergo their first autologous transplant screened for participation. Fifty-four patients, or 45% of the total screened, were found ineligible; screened patients were most commonly ineligible due to being outside study timeline (n = 20, 37%), deciding not to undergo HCT (n = 10, 18.5%), or receiving an outpatient transplant (n = 6, 11.1%) (Fig. 3). Out of the 66 eligible patients approached, 42 declined participation (63.6%), reporting feeling too overwhelmed with MM treatment to consent (Fig. 3). Demographic characteristics of the participants are shown in Table 3.

**Table 3:** Sample characteristics.

Variable	MAP-I	SHE
Age (years), mean	61	59
Range	40–74	40–78
Sex, %		
Female	31 (n = 4)	55 (n = 6)
Male	69 (n = 9)	45 (n = 5)
Race, %		
White	85 (n = 11)	73 (n = 8)
Black/African American	15 (n = 2)	27 (n = 3)
Non-Hispanic, %	100 (n = 13)	100 (n = 11)
Body Mass Index		
< 18.5	0	0
18.5–24.9	3	3
25–29.9	7	4
≥ 30	2	2
	Missingness = 1	Missingness = 2
Smoking History, %	7 (n = 1)	18 (n = 2)
KPS ≥90, %	100 (n = 11)	100 (n = 10)
	Missingness = 2	Missingness = 1
Medication Usage^a^		
Antidepressants	1	3
Opiates	2	2
Sleep Medications	1	4
None	9	6
Employment Status		
Full Time	5	2
Part Time	1	1
Unemployed	1	3
Retired	6	3
		Missingness = 2
Clinical response pretransplant		
Partial response	3	3
Very good partial response	9	6
Complete response	0	2
	Missingness = 1	

a Multiple responses possible.

Several amendments were sequentially implemented to increase enrollment and retention; amendments 1 and 2 resulted in an increase in patient enrollment and retention (Table 2). Before amendment 2, out of the 25 eligible patients approached, 32% enrolled (Fig. 3). Following amendment 2, out of the 41 eligible patients approached, 39% enrolled in the study (Fig. 3). Overall, 24 of 66 eligible patients consented and were randomized to either SHE (n = 11) or MAP-I (n = 13), yielding a 36.4% enrollment rate (Fig. 3). Of the 11 SHE group participants, 5 withdrew consent due to feeling too overwhelmed, and 2 withdrew due to becoming too sick post-HCT (Fig. 3). The remaining 4 SHE participants continued with participation through day 100 post-HCT (Fig. 3). Three of the SHE participants completed all study assessment tasks (mindfulness conceptualization, PROs, biospecimen collection, actigraphy collection) and 1 SHE participant completed all study assessment tasks except for PROs at T1 and T4 and mindfulness conceptualization at T1-T2 and T4-T5 (Table 1 and Fig. 3). Of the 13 MAP-I participants, 5 withdrew consent due to feeling too overwhelmed, 4 withdrew due to being diagnosed with COVID-19 or feeling too sick post-HCT, and 1 withdrew prior to finishing the intervention videos because they did not believe in mindfulness (Fig. 3). The remaining 3 MAP-I participants continued with participation through day 100 post-HCT and completed all study assessment tasks (Table 1 and Fig. 3).

Of the 17 randomized participants who withdrew, 100% withdrew between T1 and T3 (Fig. 1). Based on qualitative exit interviews, all participants reported feeling overwhelmed by the amount of study activities required during hospitalization. Specifically, participants who withdrew from the MAP-I group reported that it was too hard to learn and practice mindfulness in the acute recovery period. When asked what intervention adjustments would make the study more manageable, participants recommended reducing the number of MAP-I/SHE videos or shortening the length of each video.

Amendment 2 allowed all participants more time to complete the videos and provided MAP-I participants with the opportunity to learn mindfulness before HCT hospitalization. Prior to amendment 2, none of the 8 participants continued with study through day 100 post-HCT (Fig. 3). Following amendment 2 of the 16 participants randomized, 7 (43.8%) continued with study through day 100 post-HCT (Fig. 3). In total, 7 of the 24 participants continued with participation through day 100 post-HCT, resulting in an overall retention rate of 29.2% (Fig. 3). Participants in both MAP-I and SHE noted that they were more likely to withdraw prior to starting the intervention videos if they were not assigned to their preferred treatment group. Additionally, although caregiver involvement was not required for participation, eligible patients whose caregivers did not support their enrollment were less likely to enroll and/or remain in the study.

All 7 participants completed their sleep logs (100%) while 3 (42.9%) completed their weekly video log. On average, participants watched 5 of 6 videos pre-HCT and attended 2 of 3 sessions with either the mindfulness instructor or CRC during HCT hospitalization. Of the 3 MAP-I participants who completed the study, all 3 completed their sleep logs (100%), 1 completed their weekly video log (33.3%), all 3 (100%) completed their Mindfulness Practice Daily Log, all 3 (100%) completed T1-T5 actigraphy collection, 2 (66.7%) completed T1-T5 biospecimen collection (1 did not complete T1 or T5 biospecimen collection), and 2 (66.7%) completed T1-T5 surveys (1 did not complete the T1 survey). Adherence to actigraphy wear was defined as wearing the device for at least 7 consecutive days at T1 and T3-T5, and for 14 consecutive days at T2. MAP-I participants watched an average of 5 videos pre-HCT and attended an average of 2 sessions with the mindfulness instructor. Of the 4 SHE participants who completed the study, all 4 completed their sleep logs (100%), 2 completed their weekly video log (50%), 1 (25%) completed their SHE Practice Daily Log, all 4 (100%) completed T1-T5 actigraphy collection, 3 completed T1-T5 biospecimen collection (1 did not complete T5 biospecimen collection), and all 4 (100%) completed T1-T5 surveys. SHE participants watched an average of 5 videos pre-HCT and attended an average of 3 sessions with the CRC.

## 4 Discussion

The current RCT evaluated the feasibility of implementing a virtual MBI targeting sleep disruption in the peri-HCT period. Findings highlight both challenges and opportunities associated with enrolling and retaining peri-transplant MM patients in a virtual MBI sleep intervention. While the enrollment rate of 36.4 % exceeded the initially predicted 35%, the final retention rate of 29.2% did not meet the goal of 85 %, with the primary obstacle to retention being patients feeling too overwhelmed to continue while undergoing HCT. These results inform future studies targeted at implementing behavioral interventions in the peri-transplant period.

All amendments were introduced to improve retention (Table 2). While there was modest improvement with each amendment, the most meaningful change occurred when the video viewing timeline was altered to have participants watch all 6 videos pre-HCT (amendment 2, Table 2) as opposed to 2 videos pre-HCT and 4 videos during hospitalization. This amendment provided participants with more time to learn mindfulness skills before hospitalization and reduced study workload during hospitalization, increasing retention from 0 to 43.8%. While amending the video timeline improved retention, some patients reported feeling too sick or fatigued post-HCT to continue with the study, which presented another barrier to study engagement during the targeted timeframe. The final 29.2% retention rate suggests that the peri-transplant period may not be the most opportune time to learn a new skill.

While investigations from Bauer-Wu et al. (2008) and Compernolle and Sledge (2020) support the feasibility of a peri-transplant MBI, both studies conducted single-arm trials that delivered the MBI to HCT recipients in-person, presenting two notable differences compared to the present study's methodology [5,23]. The presence of a second treatment arm was influential, as participants reported upon withdrawal that they were more likely to discontinue the study if they were not assigned to the group they preferred. Additionally, MAP-I was delivered virtually, with all six videos and live sessions being delivered through a study iPad. This contrasts with these earlier peri-transplant MBI studies where a member of the study team delivered the intervention in person. In the present study, not only were participants expected to complete the intervention independently, but they were also responsible for completing questionnaires, lab draws, and actigraphy collection at five timepoints.

The peri-HCT period is a particularly stressful time, during which patients experience increased psychological distress [37,38]. This poses another challenge in considering this period as a timeframe within which to teach a new skill. Virtual behavioral interventions in other populations are equal to or more efficacious than in-person interventions [[39], [40], [41], [42], [43]]. However, while the virtual MAP-I intervention provides a scalable opportunity to improve sleep and reduce psychological distress in the peri-transplant period, all participants who withdrew departed between T1 (baseline) and T3 (14 days post-HCT) (Fig. 1). As previously noted, HCT is a procedure with a unique set of stressors; thus, the addition of a virtual MAP-I intervention and a considerable number of study related tasks may have contributed to participants feeling too overwhelmed to continue the study during the peri-transplant period. These findings highlight the importance of specific population consideration when investigating candidate interventions.

The challenge of effectively delivering an intervention during the peri-transplant period is not unique to MBIs. Previous studies have utilized a variety of behavioral interventions to treat fatigue and psychological symptoms of patients post-HCT, including physical exercise and music therapy [44,45]. While these studies demonstrated successful reduction in fatigue and improved quality of life, they report similarly low retention rates [44,45]. In one study investigating the effects of physical exercise on fatigue and quality of life in the acute post-HCT recovery period, only 33% of participants completed the study [44]. Similarly, a study examining a cognitive behavioral music therapy intervention on post-HCT fatigue reported that 11 out of 36 eligible patients (30.5%) completed the intervention [45]. Together, these findings, along with the present study's 29.2% retention rate, demonstrate the difficulty of implementing behavioral interventions during the peri-transplant period for HCT recipients. Future studies should explore varying the timing of behavioral interventions with this population. Specifically, future studies should investigate the implementation of MBI at time of diagnosis, as this may provide enough time for patients to learn, practice, and develop the habit of mindfulness before possible transplant.

Social support is a key factor when considering participant retention and engagement in study activities. Numerous studies have documented the positive effects of social support on maintaining retention [[46], [47], [48]]. In the current study, the study team noted that patients who received a brochure from their oncologist had increased interest in the intervention because it provided an opportunity for patients to receive encouragement from a trusted member of their care team. Participants often mentioned this having an overall positive impact on willingness to learn about and consent to the study. Additionally, patients noted caregiver influence as central to their initial willingness to consent. Others noted being encouraged by their caregiver to attend the virtual MAP-I or SHE sessions even when the participants felt sick post-HCT.

Previous literature has corroborated these findings, reporting that tangible or instrumental support may be key to enrolling and retaining adults with chronic health conditions in behavioral intervention trials [46]. In the current study, participants received intervention-related support only during the live virtual meetings with either the mindfulness instructor (MAP-I) or CRC (SHE) in the 2–3 weeks post-HCT. At this point, most of the intervention had been completed. Therefore, while early care team support was present and helpful at enrollment, decreased presence when participants were completing the videos (i.e., in the 6 weeks pre-HCT) may have contributed to lower than expected retention. Future studies may want to consider patient care team collaboration and caregiver participation when designing a virtual intervention.

The independent and virtual nature of the MAP-I intervention, as opposed to the in-person MBIs administered in the peri-transplant period by Bauer-Wu et al. (2008) and Compernolle and Sledge (2020) [5,23], compounded by the stress of undergoing HCT and completing PROs, actigraphy, and blood collection at five timepoints, may have been too overwhelming for participants to complete in the peri-transplant period. A current study underway is investigating the effect of a music therapy MBI on quality of life, symptom burden, and disease activity in the peri-transplant period, with feasibility results pending [49]. However, there are somewhat fewer required participant activities, and the music therapy MBI is delivered in person [49], similar to Bauer-Wu et al. (2008) and Compernolle and Sledge (2020). The current findings suggest that either enhanced patient support or lower patient participation burden may be important to achieve acceptable retention in peri-HCT behavioral intervention trials. Further research is needed to investigate how in-person versus virtual intervention delivery may impact participant engagement and retention.

Another potentially influential factor with regard to feasibility was age, as around 85 % of individuals diagnosed with MM in the United States are older than 65 [50]. A study investigating the relationship between psychological distress and engagement in mindfulness in a population of cancer survivors found age to be a significant modifier of this relationship and suggested that older adults (>65) are less likely to engage in mindfulness [51]. Additionally, there are also notable barriers to successful virtual implementation prominent with older adults, including increased wariness of technology and decreased confidence and experience with its use [52]. Thus, it is also possible that the average age of the patient population in the present study played a role in the overall willingness to engage with a virtual mindfulness-based intervention.

The present study is limited in several respects. First, the sample size was small, due in part to recruitment overlapping during peak COVID-19 restrictions and retention obstacles as detailed above. Second, most of our participants received initial MM treatment at local clinics and only came to the transplant center for pre-HCT oncology visits, which restricted the timing of recruitment. As a result, participants had a relatively restricted duration (approximately 4 weeks) to complete the week-long baseline assessment and watch 6 1-h videos, which may have been insufficient. The narrow recruitment and intervention windows may have contributed to participants feeling overwhelmed and withdrawing.

Sleep during the peri-transplant period is a critical component to optimizing both patient-reported and inflammatory outcomes following HCT. The data describe the challenges and opportunities associated with enrolling and retaining peri-HCT MM patients in a RCT implementing a sleep- and inflammation-focused MBI. These data note retention challenges to be considered when implementing a virtual MBI with medically ill individuals. The effectiveness and feasibility of MBIs should be investigated at other HCT time points and in other medically ill populations with careful consideration for the role of caregiver support, burden of study-related activities, mode of intervention delivery, and primary medical team study involvement.

## CRediT authorship contribution statement

**Elisabeth C. Henley:** Writing – review & editing, Writing – original draft, Visualization, Project administration, Methodology, Investigation, Formal analysis, Data curation. **Hannah A. Liphart:** Writing – review & editing, Writing – original draft, Visualization, Project administration, Methodology, Investigation, Formal analysis, Data curation. **Keayra J. Morris:** Resources, Conceptualization. **Iwalola Awoyinka:** Writing – review & editing. **Michael R. Irwin:** Resources, Methodology, Conceptualization. **Erin S. Costanzo:** Methodology, Conceptualization. **Diana Winston:** Resources, Conceptualization. **Anita D'Souza:** Methodology, Investigation, Conceptualization. **Melinda Stolley:** Methodology, Conceptualization. **Binod Dhakal:** Methodology, Investigation, Conceptualization. **Meera Mohan:** Methodology, Investigation, Conceptualization. **Marcelo C. Pasquini:** Methodology, Investigation, Conceptualization. **Steven W. Cole:** Methodology, Conceptualization. **Erin S. Doerwald:** Resources, Methodology, Investigation. **Peyton C. Bendis:** Writing – review & editing. **Kelly E. Rentscher:** Writing – review & editing. **Meredith E. Rumble:** Methodology, Conceptualization. **Aniko Szabo:** Methodology, Conceptualization. **Sridhar Rao:** Methodology, Conceptualization. **Jennifer M. Knight:** Writing – review & editing, Writing – original draft, Validation, Supervision, Resources, Project administration, Methodology, Funding acquisition, Formal analysis, Data curation, Conceptualization.

## Ethics approval and consent to participate

The protocol was approved by the Medical College of Wisconsin's Institutional Review Board prior to patient recruitment and consent. The clinical trial is registered at ct.gov as NCT04271930. All participants provided informed consent in writing according to the MCW IRB-approved protocol.

## Consent for publication

Not applicable.

## Availability of data and materials

The datasets used and/or analyzed in the current study are available from the corresponding author on reasonable request.

## Funding

This work was funded through the Medical College of Wisconsin Cancer Center Team Science Award and the Laura Gralton Philanthropic Fund.

## Declaration of competing interest

The authors declare the following financial interests/personal relationships which may be considered as potential competing interests:Dr. Anita D'Souza declares institutional funding from Abbvie, Caelum, Janssen, Novartis, Prothena, and Regeneron; advisory board and consulting fees from BMS, Janssen, Pfizer, and Prothena; service to American Society of Hematology. All remaining authors do not report any competing interests.

## Data Availability

Data will be made available on request.

## GLOSSARY

***CRC***: Clinical research coordinator
***HCT***: Hematopoietic stem cell transplantation
***IRB***: Institutional Review Board
***MAPs***: Mindfulness Awareness Practices
***MAP-I***: Mindfulness Awareness Practices for Insomnia
***MBI***: Mindfulness-based intervention
***MCW***: Medical College of Wisconsin
***MM***: Multiple Myeloma
***RCT***: Randomized controlled trial
***SHE***: Sleep Health Education
